# Single application of topical doxycycline in management of recurrent aphthous stomatitis: a systematic review and meta-analysis of the available evidence

**DOI:** 10.1186/s12903-020-01220-5

**Published:** 2020-08-24

**Authors:** Sadeq Ali Al-Maweri, Esam Halboub, Sajna Ashraf, Ahmed Y. Alqutaibi, Nashwan Mohammed Qaid, Kamila Yahya, Mohammed Nasser Alhajj

**Affiliations:** 1 Department of Oral Medicine and Diagnostic Sciences, AlFarabi Colleges for Dentistry and Nursing, Riyadh, Saudi Arabia; 2 Department of Oral Medicine, Oral Pathology and Oral Radiology, Faculty of Dentistry, Sana’a University, Sana’a, Yemen; 3 Department of Maxillofacial Surgery and Diagnostic Sciences, College of Dentistry, Jazan University, Jazan, Kingdom of Saudi Arabia; 4 Department of Prosthodontics, Taibah University, AlMadina AlMonawara, Saudi Arabia; 5 Department of Restorative Dental Sciences, AlFarabi Colleges for Dentistry and Nursing, Riyadh, Saudi Arabia; 6 Tooth Corner Dental Offices, Mississauga, ON Canada; 7 Department of Prosthodontics, Faculty of Dentistry, Thamar University, Dhamar, Yemen

**Keywords:** Recurrent aphthous stomatitis, Single application, Topical doxycycline, Efficacy

## Abstract

**Background:**

Recurrent aphthous stomatitis (RAS) is a highly prevalent oral mucosal disease. The management of RAS is quite challenging, and as yet, there is no definitive cure. The present systematic review and meta-analysis assessed the efficacy of a single application of topical doxycycline for the management of RAS.

**Methods:**

A comprehensive online search of PubMed, Scopus, Embase, and Web of Science databases was conducted to identify all relevant studies published up to March 31, 2019. All randomized clinical trials that assessed the efficacy of a single application of topical doxycycline in the management of RAS were included. Primary outcome measures were pain scores and/or healing time, while secondary outcomes included the associated side effects. RevMan 5.3 software was used for data analysis.

**Results:**

Five clinical trials fulfilled the eligibility criteria, three of which were included in the meta-analysis. All of the included studies found doxycycline effective in alleviating signs and symptoms of RAS. The results of the pooled 3 studies revealed a statistically significant decrease in the healing time in favor of the doxycycline group as compared to the control groups (I^2^ = 51%; MD: -1.77, 95% CI: − 2.11, − 1.42, P <0.00001); however, the results showed no significant differences between doxycycline and control groups with regard to pain reduction (I^2^ = 96%; MD: -1.28, 95% CI: − 2.83, 0.27; *P* = 0.11).

**Conclusion:**

Although still inconclusive, the available evidence suggests that a single application of topical doxycycline might be effective for reducing signs and symptoms of RAS. However, owing to the limited number of the included studies, further well-designed clinical trials with adequate sample sizes are required to discern the clinical efficacy of topical doxycycline in patients with RAS.

## Background

Recurrent aphthous stomatitis (RAS) - also known as recurrent aphthous ulcer, canker sores, and aphthous ulcer- is the most common oral mucosal disease, affecting approximately 5 to 25% of the general population [1, 2]. RAS is characterized by recurring painful ulcers confined mostly to the non-keratinized oral mucosa. Clinically, RAS presents as one of the three forms: minor, major and herpetiform [2]. The minor form is the most common type (comprising 85% of all RAS), and presents clinically as a painful round or oval ulcer of less than 1 cm in dimension, with a gray–white pseudomembrane that is surrounded by an erythematous halo [2–4]. The lesions usually heal spontaneously within 7–10 days without scarring [2–4]. Most affected patients usually develop more than one ulcer in each episode, and experience several episodes annually [2] Despite the high prevalence, and the extensive research on RAS, its etiology is still unclear; instead, a number of predisposing factors have been proposed including, but not limited to, genetic predilection, hematologic factors, immunologic defects, vitamins deficiencies, emotional stress, and local trauma [2, 3, 5–9].

Aside from being a self-limiting disease, RAS can cause severe pain, distress, and difficulty in eating and speaking that adversely affects the patients’ quality of life, especially in patients with high frequency of recurrent attacks [3, 4]. Certainly, owing to the unclear etiology, the management of RAS is quite challenging and as yet there is no specific therapy [10]. Indeed, the primary goal of RAS therapy is to reduce pain, accelerate healing, and reduce the frequency of recurrences. Many medications have been proposed to achieve the above mentioned goals; these include topical corticosteroids, antiseptic mouthwashes, anesthetics, analgesics, and antibiotics [3, 10, 11]. In severe cases of RAS like major ulcers and/or high frequent annuals attacks, systemic immunomodulatory medications such as systemic corticosteroids, colchicine, azathioprine, dapsone and pentoxifylline are used [2, 10]. Unfortunately, however, these medications can result in numerous adverse effects limiting their use [3, 10].

Tetracyclines have long been used for the management of RAS [11–13]. In addition to their antibacterial properties, these agents have anti-collagenase and anti-inflammatory effects, explaining their therapeutic action in the treatment of RAS [14, 15]. Doxycycline, a newer semisynthetic tetracycline, has been reported to have more potent anti-inflammatory and anti-collagenase properties than other tertracyclines [12–14]. More specifically, it effectively suppresses prostaglandin production and leukocytes activities; inhibits the gelatinolytic effects of collagenases; and downregulates the Matrix Metalloproteinase Collagenase (MMP)-an interstitial collagenase which is thought to play an important role in tissue destruction events in RAS [13, 14]. A recent systematic review has addressed the effects of systemic and topical doxycycline in the management of oral ulcerative lesions, and has found a significant improvement in the signs and symptoms associated with these lesions [16]. In context of the efficacy of a single application of topical doxycycline for the treatment of RAS, a number of clinical trials showed conflicting results [4, 13, 17–19]. However, no attempt has been made so far to systematically review and/or meta-analyze the available evidence in this regard. Therefore, this systematic review and meta-analysis aimed to evaluate the available evidence regarding the safety and clinical efficacy of a single application of topical doxycycline in the management of RAS.

## Methods

### Research question

The present systematic review was conducted following the Preferred Reporting Items for Systematic Review and Meta-Analyses (PRISMA) guidelines and according to the Participants, Interventions, Control, and Outcomes (PICO) principles [20]. The protocol of the present systematic review was registered at PROSPERO (ID: CRD42019128974). The addressed research question was: “Is a single application of topical doxycycline effective in the management of RAS?”

### Eligibility criteria

The studies were selected according to the following PICOS components as follows:

- Participants (P): Healthy individuals aged 15 years and above, diagnosed with RAS.
- Intervention (I): Single dose of topical doxycycline.
- Comparator (C): Any medical interventions or placebo controls.
- Outcomes (O): The primary outcomes included reduction of pain and/or healing time. The secondary outcomes included: 1) side effects of the intervention and 2) adhesive retention time.
- Study design (S): Randomized control trials (RCTs) and controlled clinical trials.

Uncontrolled clinical trials, case series, case reports, animal studies, review papers, editorials, letters to the editor, commentary and monographs were excluded.

### Search strategy

A literature search of PubMed/MEDLINE, Embase, Scopus, and Web of science was conducted in April, 2019 to identify all relevant studies published up to 31 March 2019, using various combinations of the following keywords: doxycycline, aphthous stomatitis, recurrent aphthous stomatitis*,* recurrent aphthous ulcers, recurrent oral ulcers, and canker sores. The reference lists of the selected articles were also hand-searched for additional studies. Titles and abstract of all identified articles were screened by two independent co-authors (SA and NMQ), and irrelevant studies were excluded. Full-texts of the potentially relevant studies were obtained and thoroughly evaluated by the two authors; disagreements, if any, were resolved via consensus.

### Quality assessment

Two independent co-authors (SA and NMQ) evaluated the methodological quality and the risk of bias of each included study according to the revised recommendation of the CONSORT statement tool [21]. Based on six domains (sample size estimation, randomization, blinding of participants and personnel, inclusion/exclusion criteria, comparable baseline values, completeness of follow-up, and statistical analyses), the overall estimation risk of bias was calculated for each selected study, and rated as follows: low, all criteria met; moderate, one or more criteria partly met; or high, one or more criteria were not met.

### Data extraction

For each study the following data were extracted: authors, year and country of the study, study design, types of interventions, and description of the participants (sample size, mean age, and gender), follow-up period, clinical variables (pain and healing time and side effects), drugs formulation, and the main outcomes. For meta-analyses, the means and standard deviations of the parameters of interests (primary outcomes) were extracted.

### Statistical analysis

Statistical analysis was performed using Review Manager (RevMan) Version 5.3. Copenhagen: The Nordic Cochrane Centre, the Cochrane Collaboration, 2014. The meta-analysis was conducted by measuring the mean difference between the groups along with 95% confidence intervals (CI). A *P-* value < 0.05 was considered statistically significant. Heterogeneity was evaluated using Chi-square test and the I^2^ statistics. Fixed-effects model was used for low/moderate heterogeneity while random effect model was applied for significant heterogeneity (*P* < 0.10 and I^2^ was higher than 50%).

## Results

### Study selection

**Fig. 1:**
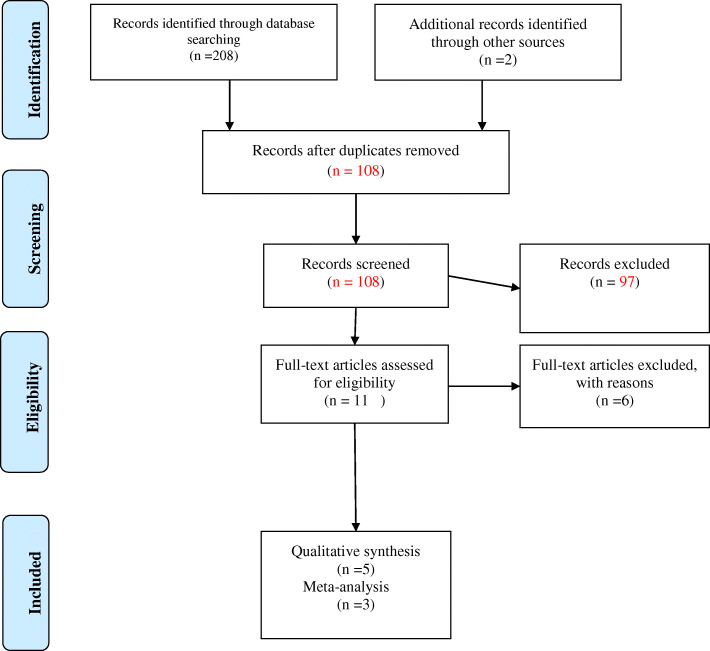
Flow-chart of methodology according to PRISMA guidelines

Online search yielded a total of 208 studies. Two additional studies were identified through hand-search, summing up to a total of 210 articles (Fig. 1). After duplicates removal (102 articles), 108 publications remained. After screening the titles and abstracts, 97 studies were found to be irrelevant, and were thus excluded. A full-text review was conducted on the remaining 11 studies, and 6 were excluded for various reasons: two used multiple doses of doxycycline [12, 15]; two were letters to the editor [22, 23]; one was in-vitro study [14]; and one [24] was a conference abstract. The remaining five studies [4, 13, 17–19] fulfilled the eligibility criteria and were further processed for data extraction. Of these, only three studies [4, 17, 19] were eligible for the meta-analysis (Fig. 1).

### General characteristics of the included studies

Five randomized clinical trials (RCT) comprising 201 patients (86 in the intervention group and 115 controls) were included [4, 13, 17–19]. Four studies were conducted in India [4, 17–19] and one was in Finland [13]. Number of subjects ranged from 30 to 50 subjects, with mean ages ranging from 15 to 64 years. The follow-up period ranged from 7 to 10 days (Table 1). All studies included patients with minor RAS. Diagnosis of RAS was based on clinical features and history of recurrent minor ulcers. The duration of RAS at the time of recruitment was reported by three studies [4, 18, 19], which ranged between 10 h and 72 h. However, no information was given about RAS duration (before intervention) by the other two studies [13, 17].

**Table 1:** General characteristics of the included studies

Author (year)	Country	Intervention	Controls	Sample size test/control	Gender	Mean age (range)	Type of RAS	Follow-up(days)
Sharma et al (2018) [18]	India	Topical Doxycycline	G1: 5% Amlexanox; G2: 0.1% Triamcinolone acetonide G3: Benzocaine gel G4: placebo gel	10/40	50%	26.5	Minor	10
Thriveni et al, (2018) [17]	India	Topical Doxycycline	Placebo 100 mg (starch, lactose, sodium starch glycolate, talc, magnesium stearate)	20/20	NA	NA	Minor	10
Chandak et al, (2017) [19]	India	Topical Doxycycline	0.1% triamcinolone acetonide paste	15/15	M = 14 F = 16	15–40	Minor	7
Vijayabala et al. (2013) [4]	India	Topical Doxycycline	Placebo 100 mg (starch, lactose, sodium starch glycolate, talc, magnesium stearate)	25/25	M = 30 F = 20	25.14	Minor	10
Ylikontiola (1997) [13]	Finland	Topical Doxycycline	Placebo (calcii gluconase)	16/15	M = 4 F = 27	23–63	Minor	10

*F* Female, *M* Male, *NA* Not Available

### Intervention and control groups

In all included studies, a single dose of topical doxycycline was applied. The efficacy of doxycycline was compared with placebo in three studies [4, 13, 17]; 0.1% triamcinolone acetonide in one study [19]; and with four control groups in one study [18]: 5% amlexanox, 0.1% triamcinolone acetonide, 20% Benzocaine gel, and placebo gel (Table 2).

**Table 2:** Drug formulation and the main outcomes of the included studies

Study	Formulation and dose	Outcome measures	Adverse effects	Main outcomes
Sharma et al (2018) [18]	Topical, Doxycycline Hyclate 100 mg was crushed and mixed with denture adhesive and saline	Pain Size of the ulcer Erythema	None	On day 4, Doxycycline and Triamcinolone acetonide groups showed better efficacy in reducing pain and ulcer size compared to other groups, but without any significant differences between the two groups.
Thriveni et al, (2018) [17]	Topical, Doxycycline Hyclate 100 mg was crushed and mixed with denture adhesive and saline	Pain Healing time	NA	Doxycycline hyclate showed a significant reduction in pain at follow-up visits as compared to placebo group (*P* < 0.001).
Chandak et al, (2017) [19]	Topical, Doxycycline Hyclate 100 mg was crushed and mixed with denture adhesive and saline	Pain Healing time	None	Healing of the ulcer was significantly faster in doxycycline group compared to triamcinolone ointment (*P* < 0.01). However, there was no a significant difference in pain reduction between the two groups (*P* > 0.05).
Vijayabala et al. (2013) [4]	Topical, Doxycycline Hyclate 100 mg was crushed and mixed with denture adhesive and saline	pain Healing time	8 patients reported transient bitter taste	A single application of doxycycline hyclate was significantly more effective in alleviating pain and reducing healing time as compared to placebo group (*P* < 0.001)
Ylikontiola (1997) [13]	Doxycycline 150 mg was crushed and mixed with saline. One drop of isobutyl cyanoacrylate as adhesive	Pain	NA	A significant reduction in pain in doxycycline group from day 2 to day 7 as compared to control group (*P* < 0.05).

*NA* Not Available

### Formulations and dose

Four studies [4, 17–19] used 100 mg Doxycycline Hyclate and one study [13] used 150 mg Doxycycline. In all included studies, doxycycline was crushed and mixed with saline. For retention of the remedy, four studies [4, 17–19] used denture adhesive while one study [13] used isobutyl cyanoacrylate (Table 2).

### Outcome measurements

All included studies [4, 13, 17–19] evaluated the efficacy of Doxycycline in reducing pain using the Visual Analog Scale (VAS). Three studies evaluated the healing time [4, 17, 19], and only one study evaluated the ulcer size [18].

## Results

### Qualitative results

All included studies found single application of doxycycline to be effective in reducing signs and symptoms of RAS [4, 13, 17–19].

#### Reduction of pain

All the included studies reported on this outcome; three studies [4, 13, 17] reported a significant reduction of pain with doxycycline intervention compared to placebo. Two studies [18, 19] found doxycycline as effective as 0.1% triamcinolone acetonide in reducing pain (Table 2).

#### Healing time

Three studies reported on this outcome [4, 17, 19], and revealed a significant decrease in the healing time with doxycycline intervention compared to the control comparators (placebo in two studies and 0.1% triamcinolone acetonide in one study).

#### Adverse effects and adhesive retention time

Four studies [4, 17–19] reported on the adverse effects. Three studies [17–19] did not find any side effects; one study [4] reported that 8 patients in doxycycline group suffered a transient bitter taste, but no side effects were reported among the placebo group. One study did not provide any information about the side effects [13]. With regards to the mean adhesive retention time, only two studies reported on this outcome, and ranged from 2.1 to 3.5 h [4, 13].

### Meta-analysis results

Only 3 studies were eligible for the meta-analysis [4, 17, 19]. The remaining two studies [13, 18] were excluded from the statistical analysis because of data unavailability. We confirm that we contacted the authors many times requesting the missing data, but unfortunately they failed to supply the necessary data.

#### Reduction of pain

**Fig. 2:**
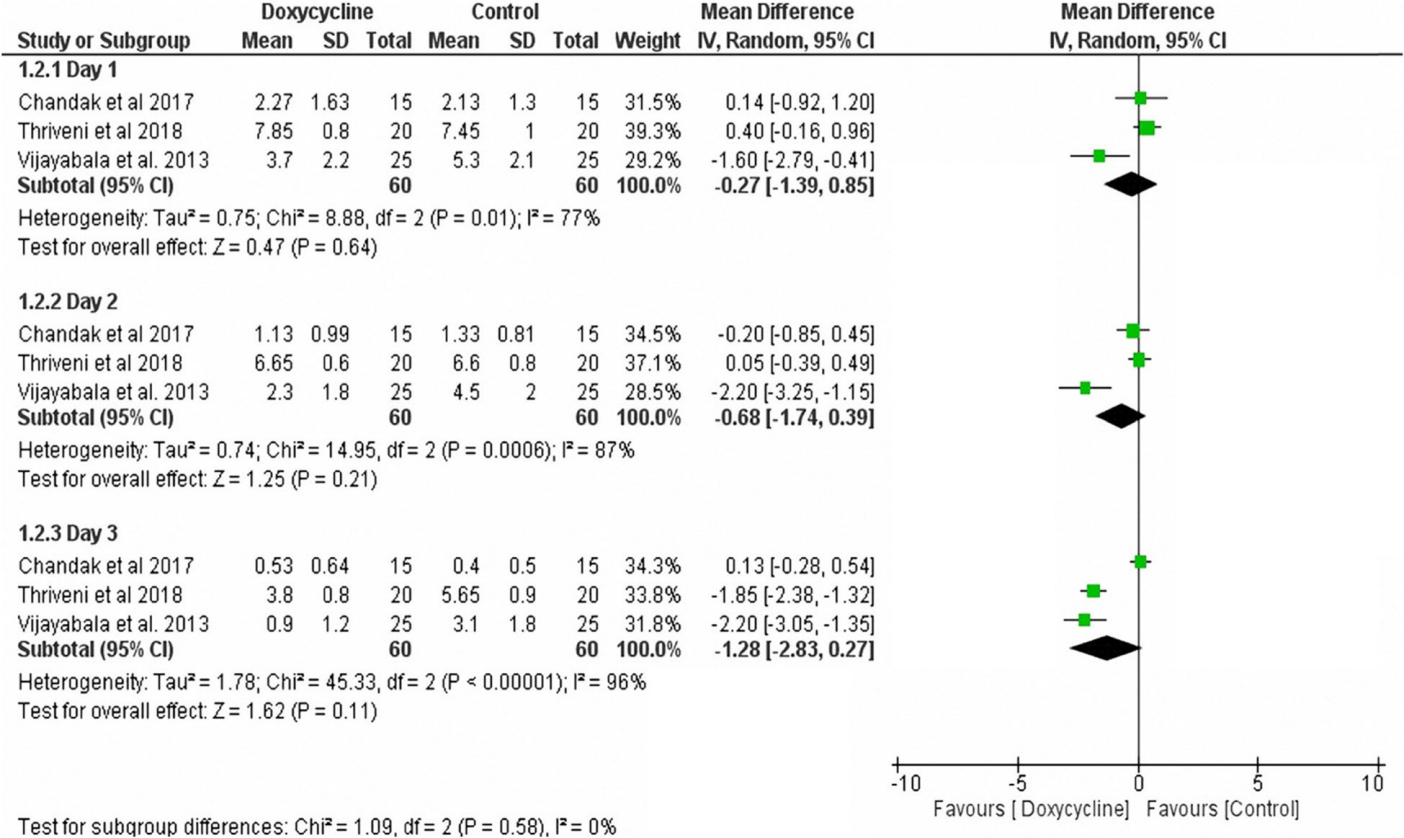
Meta-analysis of pain scores

The pooled data of the three trials [4, 17, 19] showed no significant differences in pain reduction between doxycycline intervention and the comparator interventions at day 1. At day 2 and 3, doxycycline showed better results, but again the differences were not statistically significant (Fig. 2).

#### Healing time

**Fig. 3:**
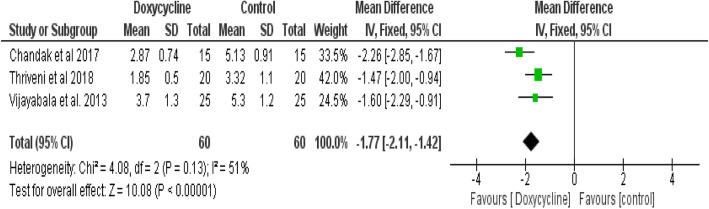
Meta-analysis of healing time

The pooled data of three trials [4, 17, 19] showed statistically significant less healing time in favour of Doxycycline intervention compared to the control interventions (I^2^ = 51%; mean difference (MD): -1.77, 95% CI: − 2.11, − 1.42, P <0.00001) (Fig. 3).

### Quality of the included studies

A summary of the risk of bias for the included studies is presented in Table 3. Three studies [13, 17, 19] showed a high risk of bias, and only two studies [4, 18] showed a moderate risk of bias. The most common methodological insufficiencies were: lack of sample size calculation, improper randomization, and absence of/inadequate blinding (Table 3).

**Table 3:** CONSORT-based quality analysis of the included studies

Study	Sample size (0–2)	Randomization (0–2)	Inclusion/ exclusion criteria (0–1)	Follow up (0–1)	Baseline (0–2)	Masking (0–2)	Statistical analysis (0–2)	score	Total Risk of Bias
Sharma et al. (2018) [18]	1	2	1	1	2	2	2	11	moderate
Thriveni et al, (2018) [17]	0	1	1	0	2	1	2	8	High
Chandak et al, (2017) [19]	0	1	1	0	2	0	1	6	High
Vijayabala et al. (2013) [4]	1	1	1	1	2	1	2	9	moderate
Ylikontiola et al. (1997) [13]	0	1	1	1	2	1	2	10	high

## Discussion

A number of clinical trials have investigated the efficacy of a single application of topical doxycycline in comparison to other treatment modalities in reducing signs and symptoms of RAS [4, 17, 19]. Therefore, the present systematic review was conducted to summarize the available evidence regarding the clinical efficacy of a single application of topical doxycycline for the management of RAS.

Overall, the qualitative analysis of all included studies showed that a single application of topical Doxycycline is effective in reducing pain and accelerating the healing time. Additionally, the meta- analysis results revealed a significant decrease in the healing time in favor of the Doxycycline group. Nevertheless, the results showed an insignificant difference between doxycycline and control groups with regard to pain reduction. Generally, the results of this review should be read with caution, given the limitations highlighted at the end of this section.

The main outcomes assessed in this review were reduction of pain and healing time of the ulcers. RAS can cause severe pain and distress that can severely impact patients’ quality of life [3]. All of the included studies assessed the efficacy of doxycycline in pain reduction using VAS, a well-known and very reliable tool for subjective assessment of pain. The results showed that doxycycline was superior to placebo but comparable to corticosteroids in reducing pain. The significant reduction in pain immediately after the treatment (starting day one after treatment) suggests a quick and potent analgesic action of topical doxycycline in patients with RAS. In line with that, the pooled data results have showed superiority of doxycycline in reducing the time required for ulcer healing. The mechanism behind the efficacy of doxycycline in reducing pain and shortening the ulcers healing time can be explained largely by its nonmicrobial properties- potent anti-inflammatory and anti-collagenase properties- in addition to its antimicrobial properties [14, 15]. Doxycycline has been shown to inhibit prostaglandin production, suppress leukocytes and inhibit MMP, thus promoting healing of the ulcer with subsequent reduction in pain [11, 13–15]. The results of the present review corroborates previous studies that found that subantimicrobial doses of systemic or topical doxycycline is highly effective in reducing pain, duration and frequency of RAS [12, 15].

One important aspect of RAS management is related to the side effects of the therapeutic agents, especially corticosteroid, the most commonly used agents. Long term use of corticosteroids, topically or systemically, is associated with serious complications like mucosal thinning, secondary candida infections, and adrenal insufficiencies, among others [3, 10, 25]. The present review revealed that the topical use of doxycycline was safe and well-tolerated by the patients without any serious adverse effects. Another important drawback of the current RAS therapeutic modalities is related to the patients’ compliance, particularly in patients with long-standing and highly frequent episodes, where the patient needs to apply the medication multiple times daily for several days. It is worth mentioning here that the single application of topical doxycycline is highly advantageous in this regard compared to other medications, resulting in a better compliance and satisfaction. In addition to the side effects and patients’ compliance, the cost of treatment is another limitation of the current therapies, especially in patients with a low socioeconomic background. Hence, a safe, efficacious, and cost- effective remedy like topical doxycycline can be considered as a good therapeutic candidate for RAS.

The present systematic review, the first of its kind, has shown promising effects of a single application of topical doxycycline in the management of RAS. However, this review has certain limitations that should be taken into account. The first limitation is related to the limited number of the included studies, and the considerable heterogeneity among the included studies with respect to the comparator group, outcome measures, duration of the ulcers, and the age of the patients. Another important limitation is related to the poor quality of the included studies reflected by the high risk of bias due to the inadequate blinding, improper randomisation and/or small sample sizes. Additionally, the short follow-up period is an obvious shortcoming of the included studies, and thus no attempt was made to assess the efficacy of doxycycline on the recurrence rate of the episodes. Therefore, further high-quality clinical trials with large sample sizes and adequate follow-up periods are required to discern the efficacy of a single application of topical doxycycline for treating RAS.

## Conclusions

Within the limitations of the available evidence drawn from the current systematic review and meta-analysis, we can conclude that a single application of topical doxycycline has a promising effect in reducing pain and accelerating healing time in patients with RAS. However, given the limited number of the included studies and low quality of these studies, further well-designed multicenter RCTs are highly warranted.

## Data Availability

The datasets supporting the findings of this article are available from the corresponding author.

## Abbreviations

RAS: Recurrent Aphthous Stomatitis
RCTs: Randomized Clinical Trials
VAS: Visual Analog Scale
MD: Mean Difference
MMP: Matrix MetalloProteinase
